# Supplementary material for: Blockade of Glycosphingolipid Synthesis Inhibits Cell Cycle and Spheroid Growth of Colon Cancer Cells In Vitro and Experimental Colon Cancer Incidence In Vivo
Source: Int J Mol Sci. 2021 Sep 29;22(19):10539. doi: 10.3390/ijms221910539 (PMC8508865; doi:10.3390/ijms221910539)
Supplement: Supplementary file 1 [file ijms-22-10539-s001.zip › ijms-1383246-supplementary.pdf]

Blockade of glycosphingolipid synthesis inhibits cell cycle and spheroid growth of colon cancer cells in vitro and experimental colon cancer incidence in vivo

Richard Jennemann, Martina Volz, Felix Bestvater, Claudia Schmidt, Karsten Richter, Sylvia Kaden, Johannes Müthing, Hermann-Josef Gröne, and Roger Sandhoff

Supporting Figures

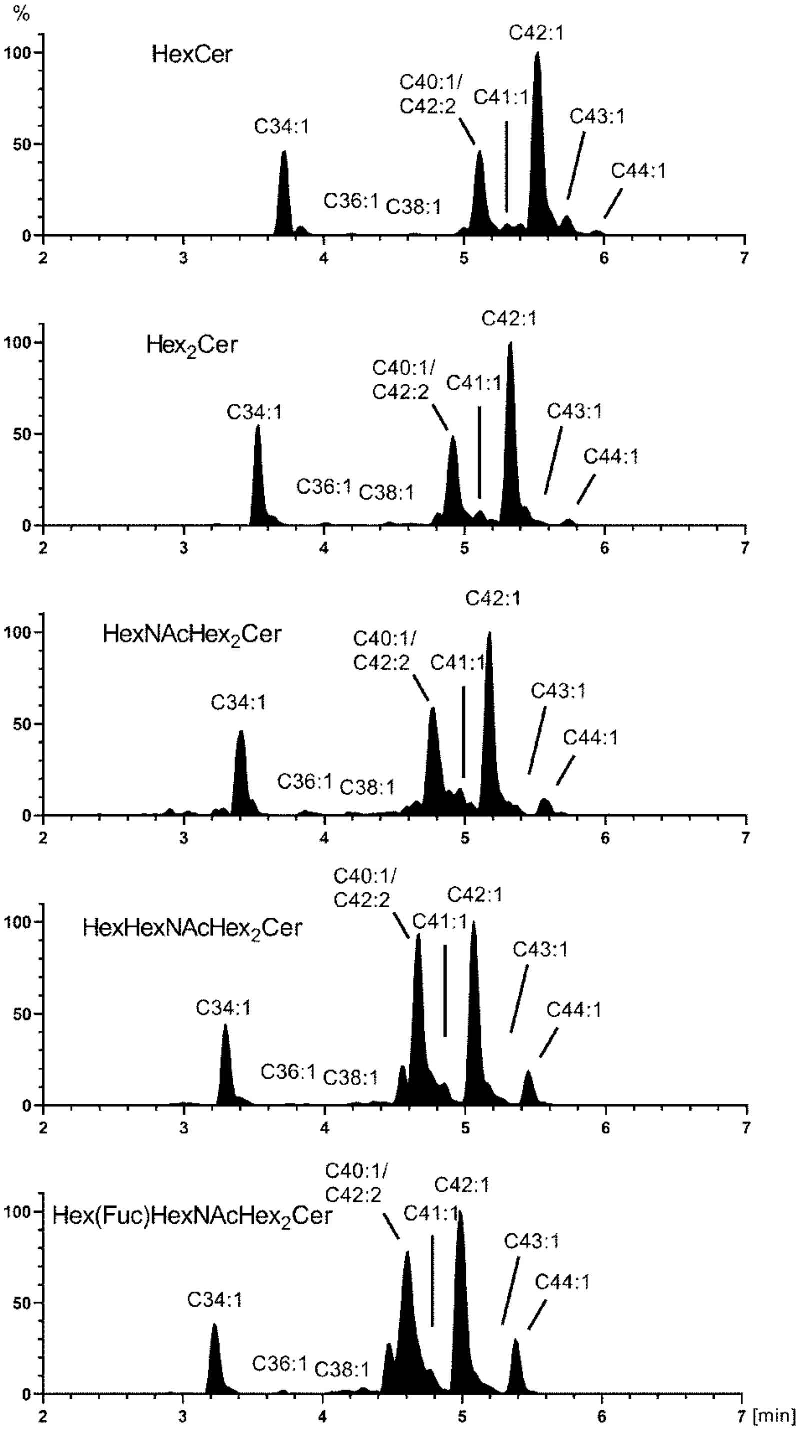

Figure S1

Ultra performance liquid chromatography (UPLC)/MS^2 elution pattern of neutral GSLs from Lovo cells. According to their migration on TLC, paired with the masses obtained from the peaks separated by UPLC correlating with: HexCer (GlcCer/GalCer), Hex_2Cer (LacCer), $HexNAcHex_2Cer$ (Lc_3Cer), $HexHexNAcHex_2Cer$ (Lc_4Cer), and $Hex(Fuc)HexNAcHex_2Cer$ (Lewis^a), the carbohydrate components of Lovo cells can be associated with lacto-series GSLs. GSLs were detected in MRM mode. Peaks were annotated for their ceramide anchor composition indicating first the number of carbon atoms (i.e. "C34") and thereafter the total number of double bonds (i.e. ":1" for one double bond).

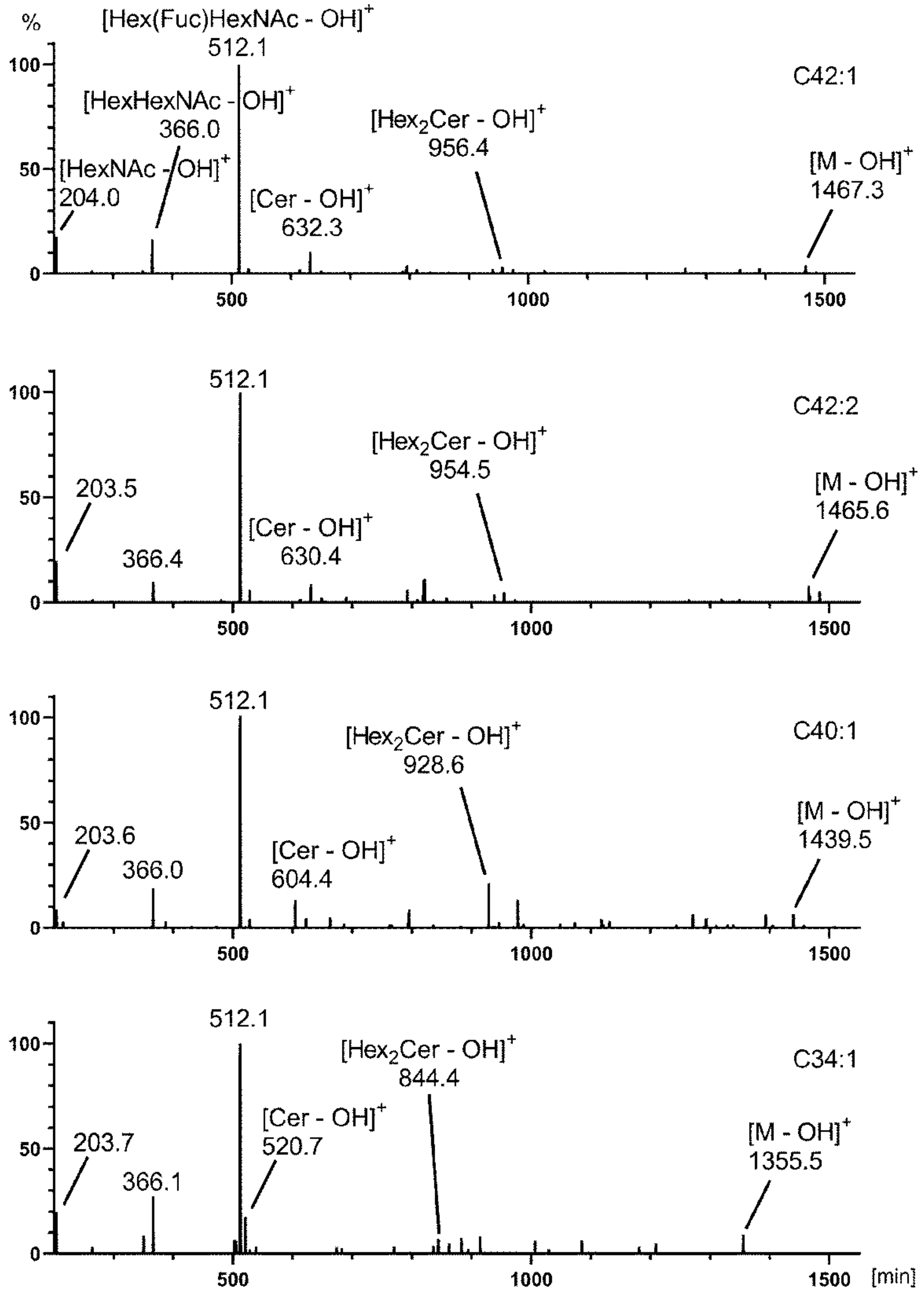

Figure S2

Fragment ion spectra of neutral component Hex(Fuc)HexNAcHex$_2$Cer (Lewis[a]) from Lovo cells. Lewis[a] with different fatty acid lengths was fragmented. The m/z 512 indicated the Hex(Fuc)HexNAc fragment of Lewis[a]. In addition, the core Hex$_2$Cer residue was present in all spectra, which depended in m/z on the ceramide anchor composition.

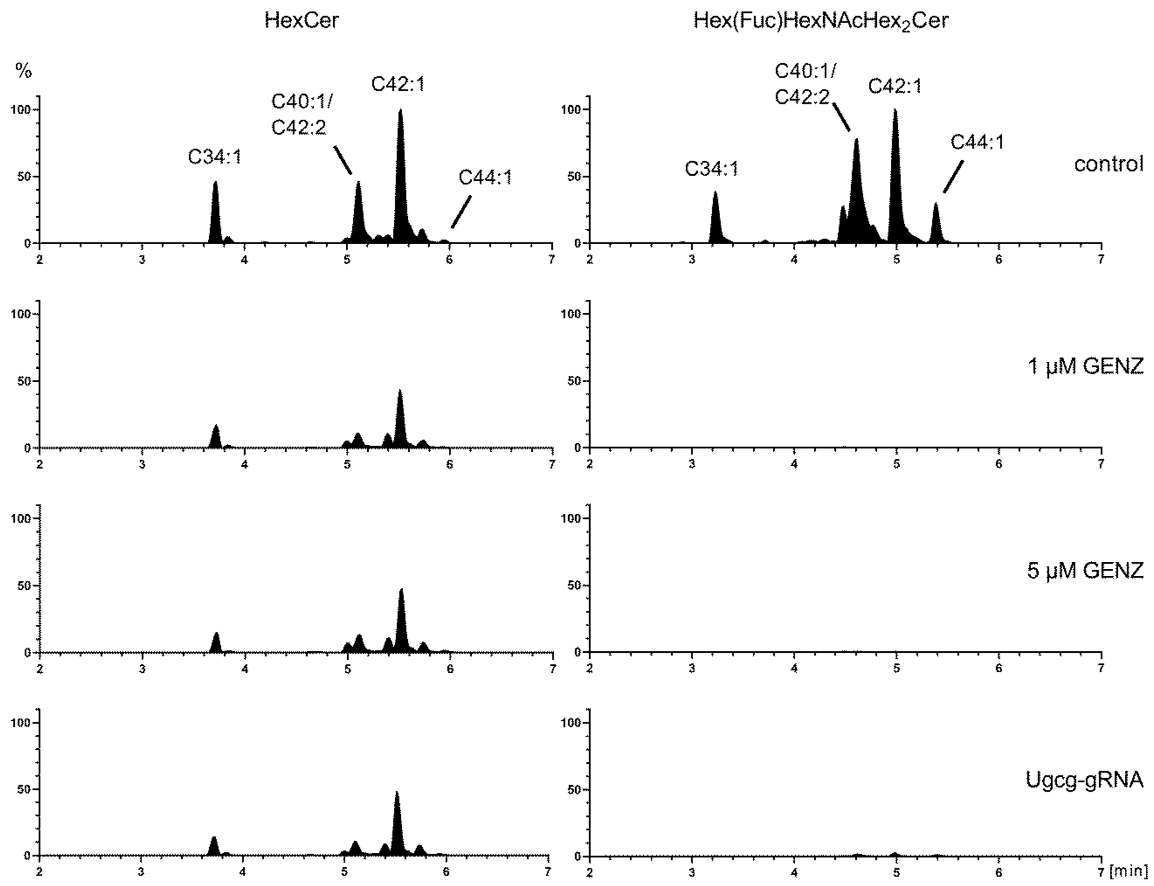

Figure S3

Semiquantitative determination of GSL-reduction in Lovo cells by C18-reversed phase UPLC/MS^2. Lovo cells were treated either with Genz or with *UGCG*-gRNA in order to disrupt GSL synthesis. GSL were detected in MRM mode and peak intensities were normalized to controls, which were set to 100%. A reduction of ~50 % could be detected within the UPLC-spectra representing the HexCer-species. The remaining HexCer could be verified to consist of GalCer by perborate separation (see Supporting Figure 5), whose synthesis was not affected by Genz-treatment or genetic *UGCG*-targeting. The GlcCer-based component Hex(Fuc)HexNAcHex2Cer (Lewis[a]), however, was almost completely depleted independent from the method of GSL-silencing.

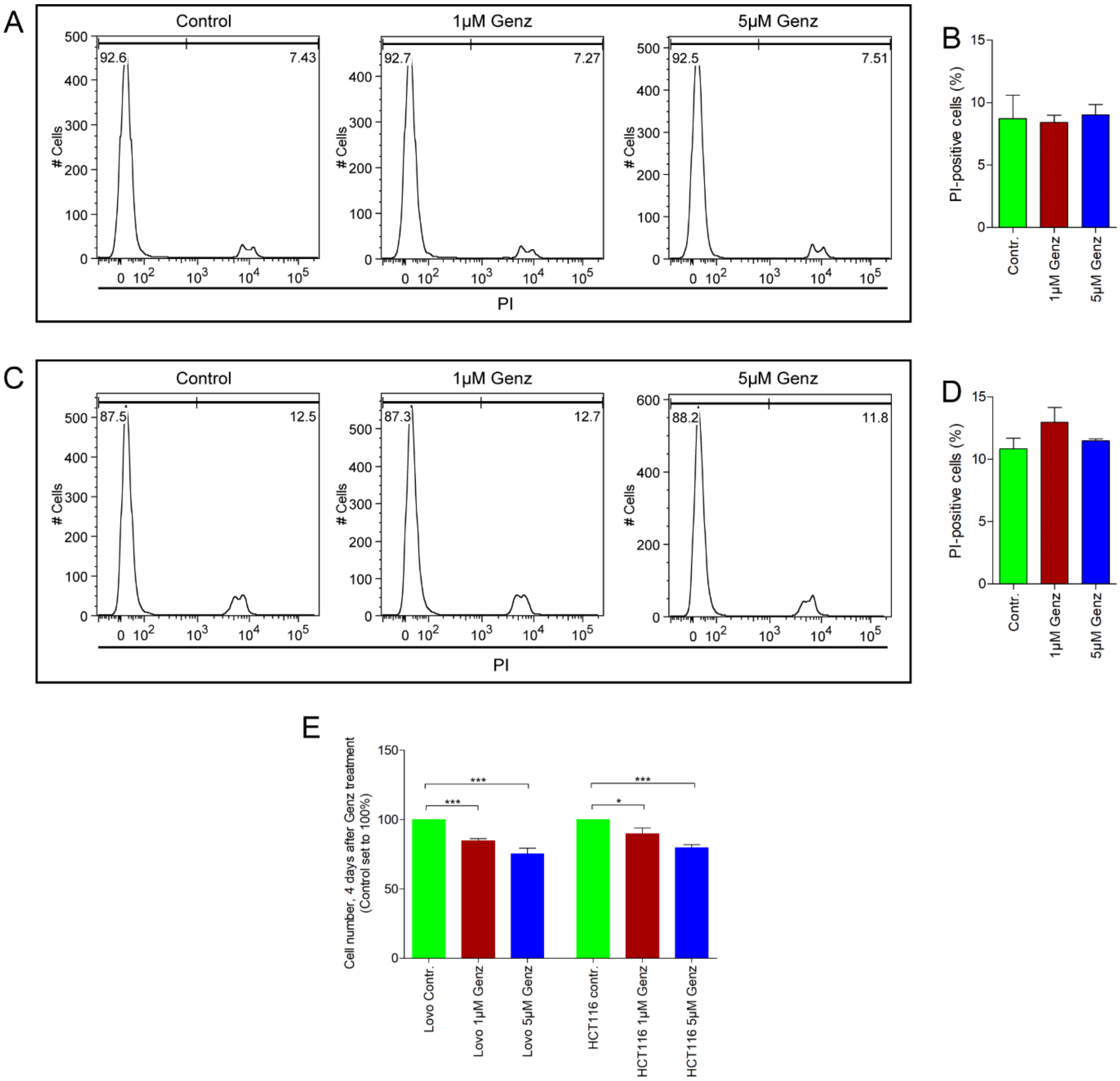

Figure S4

Genz-treatment does not induce necrosis in colon carcinoma cells but reduces proliferation. (**A** and **C**) PI staining of Lovo- (**A**) and HCT116 cells (**C**); (**B** and **D**, quantification). Lovo and HCT116 cells were treated with the indicated concentrations of Genz for six days. Cells were stained after trypsination for 30 min with propidium iodate (PI) in order to quantify dead cells. Differences in cell viability upon Genz-treatment were not detected. (**E**) Equal numbers of Lovo- and HCT116 cells were cultivated with or without the indicated concentrations of Genz and counted after four days; n=4 each. Treatment with 1 µM Genz resulted in a lower cell number, which even stronger decreased upon 5 µM Genz application indicating reduced proliferation.

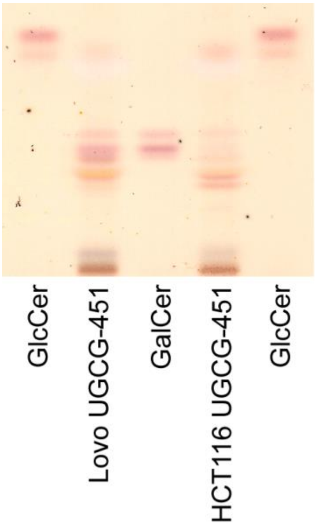

Figure S5

TLC perborate separation of GlcCer and GalCer. Neutral GSLs from *UGCG*Crispr/Cas9-treated Lovo- and HCT116 cells corresponding to 0.2mg protein were spotted on TLC and sprayed with perborate solution. GSLs were separated in $CHCl_3/CH_3OH/H_2O$/25 % NH_4OH, 62.5:30:6:0.5 by vol.. The residual GSLs, migrating on the height of hexosylceramide consisted of GalCer which was not targeted by the *UGCG*-gRNA.

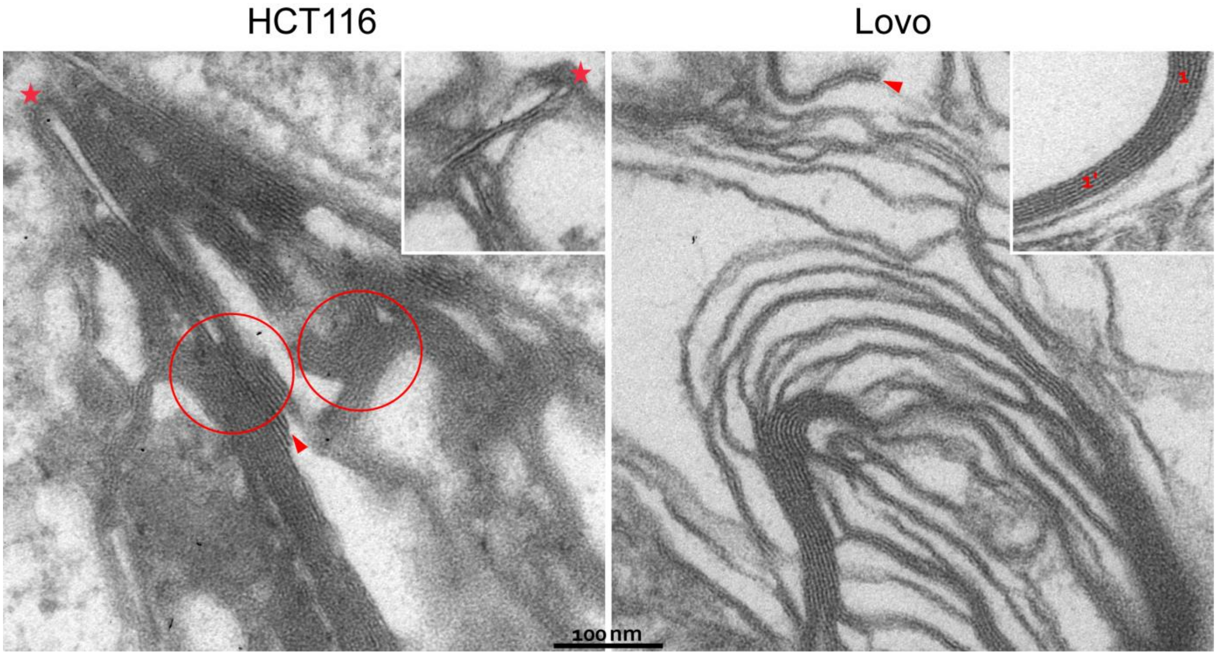

Figure S6

Genz treatment leads to unusual lipid-organization of membranes. Upon Genz-treatment, lysosomal membranes of HCT116 cells tended to form closed stacks with 3.8 nm periodic repeat (left image). Unless true myelin, bi-lamellar membranes were seen to end blindly (arrow head), to widen into blistered ends (asterisk), and to disorganize in micellar convolutions (circled area). Lysosomal membrane-stacks in Genz-treated Lovo cells had a similar periodic repeat of 4 nm (right image). Here too, blindly ending membranes were found (arrow-head), suggesting micellar sealing.

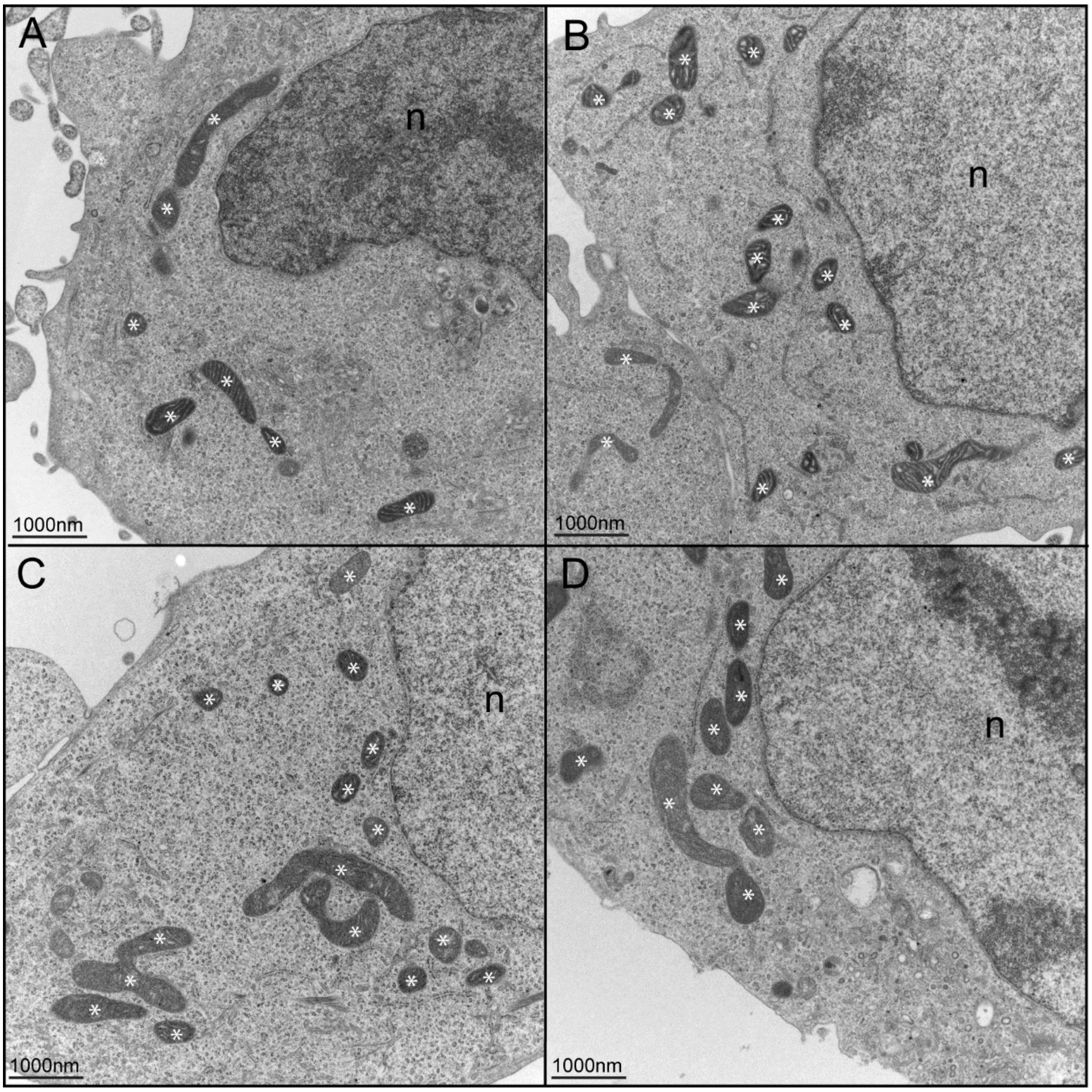

Figure S7

UGCG-inhibition of Lovo and HCT116 cells with Miglustat or deletion by Crispr/Cas9 technology does not cause lysosomal alterations. (**A** and **B**) Treatment of Lovo cells (**A**) and HCT116 cells (**B**) with 100µM Miglustat did not cause ultrastructurally visible lipid accumulation. (**C** and **D**) *UGCG*-gRNA application did also not lead to lipid storage in lysosomal compartments of Lovo- (**C**) and HCT116 cells (**D**); asterisks mark mitochondria; n, nucleus.

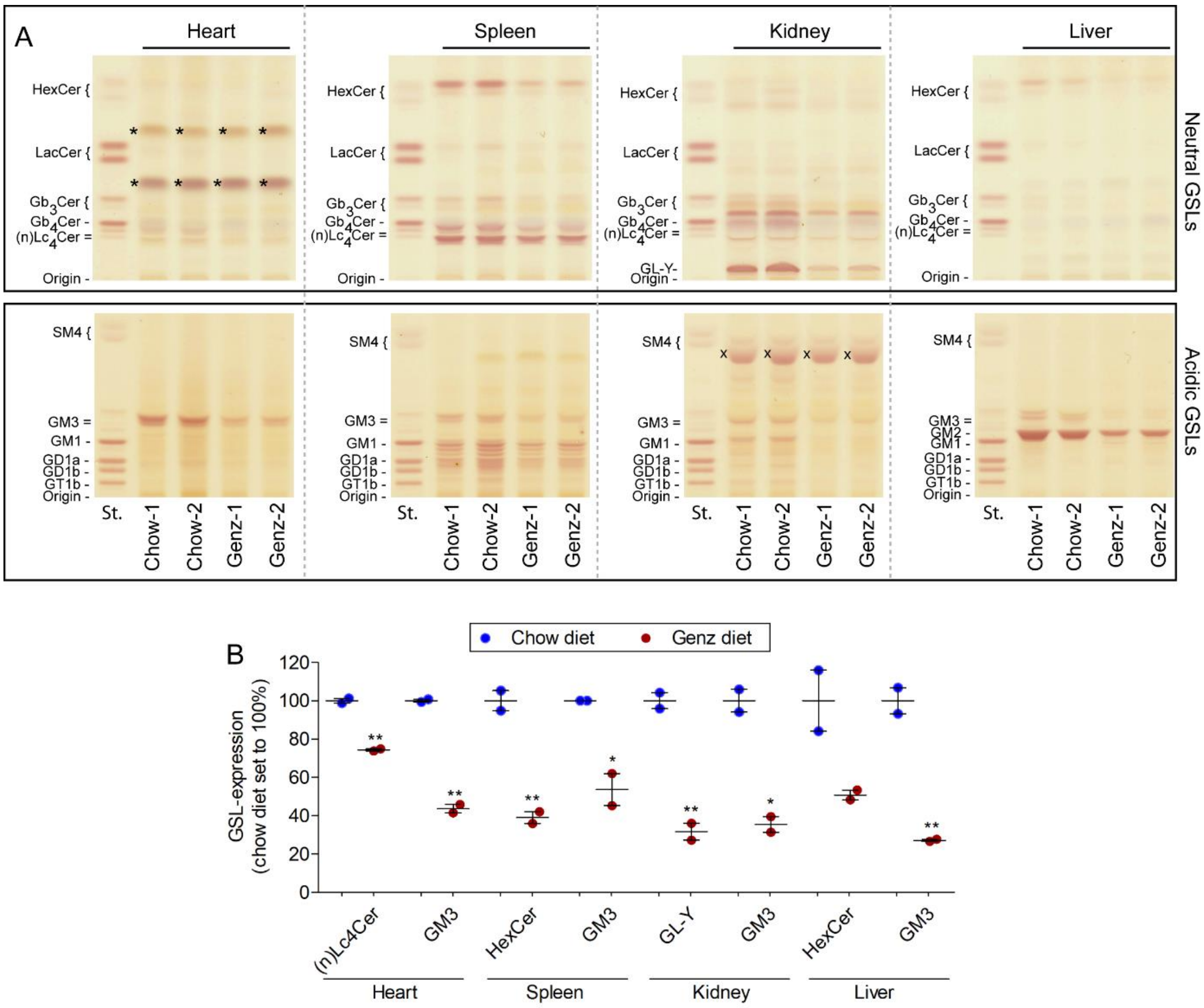

Figure S8

TLC- analysis of organs from Genz-fed mice indicates a significant reduction of GSLs. (**A**) TLC-analysis from heart, spleen, kidney, and liver showed a marked reduction of neutral and acidic GSLs upon Genz treatment. (**B**) Quantification of major neutral GSLs and GM3 within the acidic fraction by ImageJ. Genz-feeding led to decreased GM3 content of at least 50%.

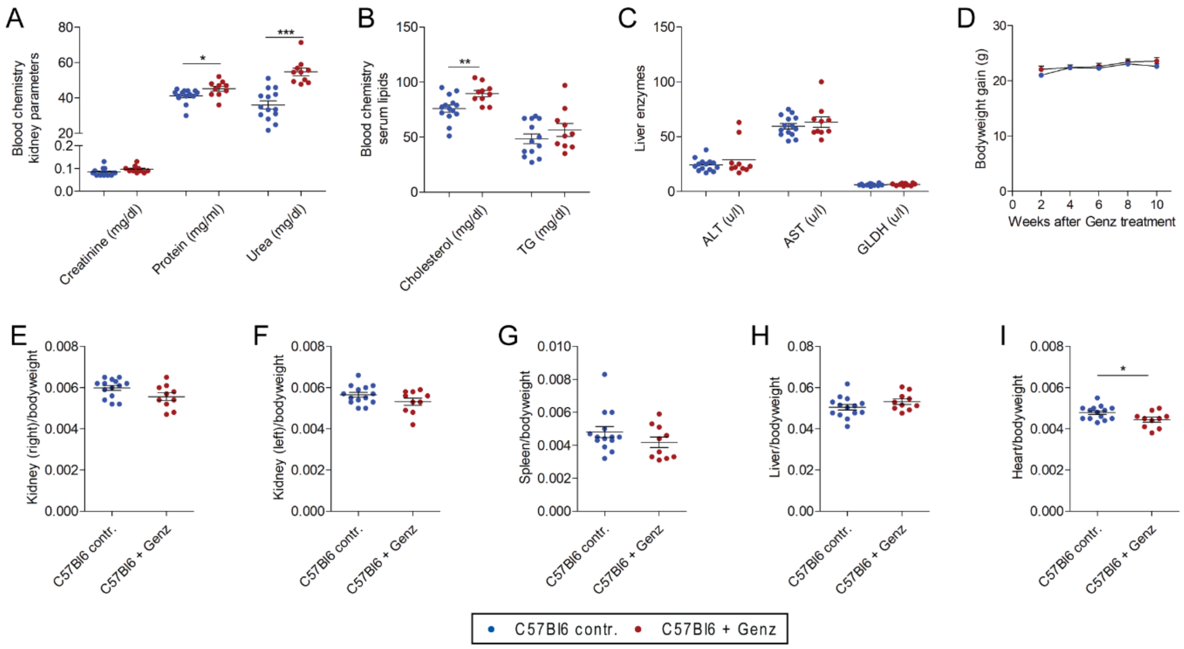

Figure S9

Genz treatment of mice induces slightly elevated serum-protein, urea and cholesterol levels. (**A** to **C**) blood chemistry. Serum-protein-, urea- (**A**) and cholesterol levels **(B**) increased slightly upon Genz-treatment. Liver parameters remained largely unaffected (**C**). (**D**) Genz-feeding did not influence bodyweight gain. (**E-I**) The organ- per bodyweight ratio was similar in treated mice and controls.
